# Extracellular vesicles as mediators of the progression and chemoresistance of pancreatic cancer and their potential clinical applications

**DOI:** 10.1186/s12943-017-0755-z

**Published:** 2018-01-05

**Authors:** Jiangdong Qiu, Gang Yang, Mengyu Feng, Suli Zheng, Zhe Cao, Lei You, Lianfang Zheng, Taiping Zhang, Yupei Zhao

**Affiliations:** 1 Department of General Surgery, Peking Union Medical College Hospital, Chinese Academy of Medical Sciences and Peking Union Medical College, No. 1 Shuaifuyuan, Wangfujing Street, Beijing, 100730 China; 2 Department of Nuclear Medicine, Peking Union Medical College Hospital, Chinese Academy of Medical Sciences and Peking Union Medical College, Beijing, 100730 China; 3 Clinical Immunology Center, Chinese Academy of Medical Sciences and Peking Union Medical College, Beijing, 100730 China

**Keywords:** Extracellular vesicles, Pancreatic cancer, Chemoresistance, Clinical applications

## Abstract

Pancreatic cancer is one of the most lethal cancers worldwide due to its insidious symptoms, early metastasis, and chemoresistance. Hence, the underlying mechanisms contributing to pancreatic cancer progression require further exploration. Based on accumulating evidence, extracellular vesicles, including exosomes and microvesicles, play a crucial role in pancreatic cancer progression and chemoresistance. Furthermore, they also possess the potential to be promising biomarkers, therapy targets and tools for treating pancreatic cancer. Therefore, in-depth studies on the role of extracellular vesicles in pancreatic cancer are meaningful. In this review, we focus on the regulatory effects of extracellular vesicles on pancreatic cancer progression, metastasis, cancer-related immunity and chemoresistance, particularly their potential roles as biomarkers and therapeutic targets.

## Background

Pancreatic ductal adenocarcinoma (PDAC, hereafter referred to as pancreatic cancer [PC]) is the seventh most common malignancy, ranking as the fourth and sixth leading causes of cancer-related death in the USA and China, respectively. Patients with PC have a 5-year survival rate of approximately 6% and a median survival rate of 6 months [1–3]. PC is expected to become the second leading cause of cancer-related death in the USA by 2030 [4]. The dismal prognosis of PC is mainly attributed to poor detection rates at early stages, rapid progression and disappointing surgical resection outcomes. Most patients with PC lack diagnostic symptoms during early stages, and the existing screening biomarkers, such as CA19–9, are not sufficient for a highly sensitive and specific diagnosis of PC [5]. When a patient is diagnosed with localized PC, surgical resection is regarded as the only potential curative treatment [6]. However, patients should expect only a 5-year survival rate of 23–26% before recurrence [7]. Unfortunately, approximately 80% of patients have reached an unresectable stage at the time of diagnosis. Chemotherapy with gemcitabine is an indispensable treatment for these patients. However, the effectiveness of chemotherapeutic drugs is often plagued by chemoresistance, worsening the outcomes of patients with metastatic disease [8, 9]. Thus, a better understanding of the underlying cellular and molecular mechanisms of PC progression and chemoresistance is urgently needed.

Extracellular vesicles (EVs), which were considered containers of cellular debris, have recently been highlighted as intercellular communication tools and mechanisms of molecular transfer [10–12]. EVs include exosomes, ectosomes, microvesicles, apoptotic bodies and oncosomes, according to their sizes and biogenesis mechanisms [13]. Many types of cells have the capacity to secrete EVs, including dendritic cells (DCs) [14], B and T cells [15], neurons [16], fibroblasts [17], stem cells [18], and cancer cells [19]. EVs are also detected in and isolated from multiple biological fluids, such as blood, urine, ascitic fluid, saliva and supernatants [20, 21]. EV-mediated intercellular communication is achieved by biologically active substances, such as proteins and nucleic acids, carried by EVs. Similarly, the interactions between cells and the tumor microenvironment are also partially mediated by EVs [22]. EVs have been shown to play an important role in the tumorigenesis, progression, metastasis and chemoresistance of various malignancies, such as breast cancer [23], hepatocellular carcinoma [24] and prostate cancer [25]. The functions of EVs in PC have also been investigated [26]. In this review, we will discuss the roles of EVs in the progression, metastasis and chemoresistance of PC as well as their promising applications as biomarkers and therapeutic targets in PC.

## Biological features of PC-derived EVs

### Morphology, biogenesis and secretion of EVs

Exosomes and microvesicles (MVs) are two main subtypes of EVs, with diameters of 30–120 nm and 120–1000 nm, respectively. Exosomes, which are typically composed of a lipid bilayer membrane surrounding a small cytosol lacking cellular organelles, were first described by Johnstone RM in 1987 during the in vitro culture of sheep reticulocytes [11, 12]. Exosomes are usually cup/disk-shaped and originate from a late endosome following inward budding of multivesicular bodies (MVBs), whereas MVs are irregularly shaped and directly formed by cell membrane shedding [27, 28]. The intraluminal vesicles (ILVs) budding from MVBs are the original forms of exosomes. Three independent pathways are involved in exosomal biogenesis: the endosomal sorting complex required for the transport (ESCRT)-dependent pathway and the ceramide-dependent pathway contribute to the formation of ILVs. Inhibition of the two pathways decreases exosomal biogenesis and secretion. While the tetraspanin-dependent pathway is responsible for selecting cargoes for exosomes [29, 30]. Exosome secretion is mediated by RAB GTPase proteins which control intracellular vesicles trafficking and docking to plasma membrane, and soluble NSF-attachment protein receptor (SNARE) complexes which allow fusion of lipid bilayer [30, 31]. Increasing intracellular Ca^2+^, lower pH in microenvironment, up-regulation of P53 protein and heparanase stimulate exosomes secretion [32–34]. Besides, the EVs released by cancer cells can be triggered by anti-cancer therapy, questioning the long-term efficacy of such treatment because EVs may promote PC progression as discussed in the context [35]. Three mechanisms are currently reported to be involved in exosome uptake by receipt cells: (1) an interaction between a surface receptor and ligand, (2) internalization through direct fusion, and (3) internalization through endocytosis [36–38].

Several methods, such as ultracentrifugation, affinity isolation, size exclusion chromatography and membrane filtration, have been applied to isolate EVs from conditioned cell culture medium and body fluids. But most published studies of EVs have employed ultracentrifugation, the “Gold Standard” for EV isolation [11]. The International Society for Extracellular Vesicles (ISEV) has provided authoritative guidance for EV isolation and purification [39]. EV detection can be realized by transmission electron microscopy (for direct imaging) and Western blotting or flow cytometry (for EV markers analysis) [40–42]. The markers used for exosome analysis include tetraspanins (including CD9, CD63, CD81, and CD82), ESCRT-associated proteins (including tumor susceptibility gene 101 (TSG101) and apoptosis-linked gene 2-interacting protein X (ALIX)), cytoplasmic proteins (heat shock protein 70 (HSP70) and HSP90), adhesion molecules (integrins), and membrane transport and fusion proteins (annexins) [30, 43, 44].

## Role of EVs in PC

**Fig. 1:**
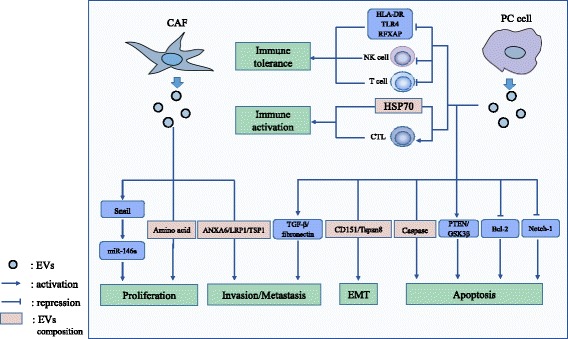
The functions of EVs in PC. The regulatory effects of EVs on cell proliferation, metastasis, and tumor-associated immunity are shown

The histology of PC is characterized by a complex microenvironment consisting of PC cells and other components including vascular endothelial cells, immune cells, fibroblasts, myofibroblasts, stellate cells and extracellular matrix(ECM). Cancer-associated fibroblasts (CAFs) promote remodeling of ECM and tumor growth. Immune cells in the microenvironment of PC have a highly immunosuppressive composition and further contribute to immune evasion [45]. The interactions between cancer cells and the tumor microenvironment are crucial steps in tumor progression [46–48], contributing to the altered metabolism and hyper-proliferation of cancer cells as well as tumor metastasis and abnormal tumor-associated immunity [46, 49, 50]. The role of EVs in PC is illustrated by these interactions across this part because these interactions are partially modulated by EVs. For example, EVs from fibroblasts promote invasive behavior and upregulate drug resistance pathways in cancer cells, whereas EVs from tumor cells reprogram normal fibroblasts into CAFs [51–53]. Here, we describe the following functions of EVs in PC: (1) regulating the proliferation of PC cells, (2) promoting PC invasion and metastasis, and (3) modulating tumor-associated immunity. Figure 1 summarizes the functions of EVs in PC.

### EVs regulate PC cell proliferation

EVs play different roles in regulating PC cell proliferation, depending on their origin. In vitro studies, exosomes released from gemcitabine-treated CAFs increased the proliferation and survival of both chemosensitive and chemoresistant PC cell lines. It was partially attributed to the increased level of Snail and its target, miR-146a, in recipient cells. Inhibition of exosome secretion from CAFs reduced PC cell proliferation and survival [54]. Furthermore, CAF-derived exosomes (CDEs) rescued the proliferation of nutrient-deprived BxPC3 and MiaPaCa-2 cells by supplying them with metabolites in a KRAS-independent manner. This effect was blocked by heparin, an inhibitor of receptor-mediated endocytosis [49]. However, EVs isolated from PC cells (SOJ-6) supernatant downregulated PC cells proliferation by activating phosphatase and tensin homolog (PTEN) and glycogen synthase kinase 3 beta (GSK-3β), thus evoking the mitochondria-dependent apoptotic pathway [55]. Decreased expression of hairy and enhancer-of-split homolog-1 (HES-1), the intranuclear target of the Notch-1 signaling pathway, also promoted the EV-induced suppression of PC cells proliferation [56]. These in vitro studies indicate EVs with different origins exert opposite effects on PC cell proliferation, which requires further elucidation. Although circulating EVs from healthy people’s serum have been approved to induce apoptosis in PC cells [57], further in vivo studies are needed to assess the effects of EVs on PC cells proliferation and tumor growth, which may indicate a possible intervention method for PC therapy.

### EVs promote PC cell invasion and metastasis

EVs modulate PC invasion and metastasis because of their regulatory effects on PC cells and the tumor microenvironment. EVs derived from pancreatic stellate cells (PSCs) and enhanced the migration of PC cells, which were decreased by an exosome inhibitor [58]. CAF-derived annexin 6A–positive (ANXA6+) EVs containing the annexin A6/LDL receptor-related protein 1/thrombospondin1 (ANXA6/LRP1/TSP1) complex increased PC aggressiveness following uptake by PC cells, and ANXA6 depletion via infection of shANXA6 in CAFs impaired tumor metastasis [59]. EVs with in vivo origins from malignant effusions of colorectal cancer, breast cancer and small lung cancer induced PC cell migration [60]. Another in vivo study found that EVs derived from PC cells contributed to the formation of the pre-metastasis niche in the liver [3]. In this study, PC-derived exosomes expressing high levels of macrophage migration inhibitory factor (MIF) fused with Kupffer cells, activated fibrotic pathways and created a proinflammatory environment. These changes supported metastasis by upregulating the expression of transforming growth factor β (TGF-β) and fibronectin as well as recruiting bone-marrow-derived cells (macrophages and neutrophils) to the liver [3]. Furthermore, the formation of a pre-metastasis niche by exosomes required the help of CD44v6 [61], a cancer-initiating cell (CIC) marker, to provide a soluble matrix in vivo and enable exosomes to transfer migratory and invasive capacity to non-CICs [61–63]. Moreover, the epithelial-mesenchymal transition (EMT) is supported by CD151−/tetraspanin 8-competent exosomes, which drives the differentiation of non-metastatic PC cells toward a motile phenotype [64]. Taken together, EVs derived from tumor cells or other cellular components of the tumor microenvironment indeed play a positive role in PC invasion and metastasis, with the assistance of different molecules.

### EVs modulate tumor-associated immunity

Cancer is characterized by impaired immune surveillance and tolerance toward cancer cells. The pancreatic tumor microenvironment is immunosuppressive because of inhibitory cytokines and recruitment of immunomodulatory cells like myeloid-derived suppressor cells (MDSCs) which suppress T-cell activation via TGFβ [45, 65]. Besides, B cells, tumor-associated macrophages (TAMs), and Tregs also play immunosuppressive roles in TME of PC by suppressing the function of cytotoxic T cells (CTLs) and CD8^+^ T cells [45]. EVs have recently been shown to play a pivotal but contradictory role in PC immunity. On one hand, EVs derived from PC cells or immune cells induce immune elimination. On the other hand, some PC-derived EVs captured by immune cells circumvent the immune response. For instance, exosomes derived from the ASML cell line inhibit the proliferation of leukocytes and reduce T cell migration, whereas they support the activation of effector lymphocytes, such as CTLs [66]. Exosomes released from cancer cells induced monocyte survival in the tumor microenvironment by regulating the mitogen-activated protein kinase (MAPK) pathway, resulting in the continuous generation of TAMs [67]. Besides, PC-derived exosomes suppress the immune system by downregulating human leukocyte antigen D related (HLA-DR) expression in monocytes and the tumor-killing capacity of natural killer (NK) cells [68, 69]. Furthermore, miR-203 and miR-212-3p are also involved in immune tolerance caused by PC-derived exosomes via decreasing the expression of toll-like receptor 4 (TLR4) and regulatory factor X-associated protein (RFXAP) in DCs [70, 71]. In contrast, the exosomal proteins with miRNA depleted via miRNA lysis and ultrafiltration activate DCs/cytokine-induced killer (CIK) cells to target PC [72]. HSP70 surface-positive exosomes derived from PC cells promote the migration and cytolytic activity of NK cells [73]. Overall, the pro- or anti-effects of EVs on tumor-associated immunity depend on differences in the EV composition and target cells, but these different functions of EVs may contribute to investigations of the use of modified EVs as a possible tumor vaccine.

## EVs participate in the formation of chemoresistance in PC

Currently, chemotherapy is an indispensable treatment option for patients with advanced pancreatic cancer. Several clinical trials have shown that adjuvant chemotherapy based on fluorouracil and gemcitabine (GEM) improved survival in patients with resected PC [74, 75]. A randomized phase III trial has proven that the non-inferiority of oral S-1, a fluoropyrimidine derivative had a higher 2-year overall survival than GEM in adjuvant chemotherapy [76]. For metastatic PC, chemotherapy includes GEM monotherapy or GEM plus novel regimens, such as erlotinib [77] and nanoparticle albumin-bound paclitaxel [78]. Besides, the FOLFIRINOX regimen which consists of oxaliplatin, folinic acid (leucovorin), irinotecan, bolus fluorouracil, and infusional fluorouracil has better chemotherapy response than GEM monotherapy in metastatic PC [79]. Unfortunately, however, chemoresistance occurs in most cases after long-term exposure to chemotherapeutics, particularly GEM, the standard chemotherapeutic agent for unresectable pancreatic cancer. Based on accumulating evidence, EVs may play a role as intercellular communicators in promoting chemoresistance in multiple cancers, including leukemia [80], glioblastoma [81], lung cancer [82], gastric cancer [83], breast cancer [84], prostate cancer [25], ovarian cancer [85] and PC [9]. The mechanisms underlying the EV-mediated chemoresistance in cancers include transferring the drug-resistance-related gene multidrug resistant-1 (MDR-1), P glycoprotein [86], survivin [87] and ubiquitin carboxyl terminal hydrolase-L1 [84] to recipient cells.

The effects of EVs on PC chemoresistance partially rely on the RNA it transfers. When incubated with GEM, PC cells upregulated the expression of miR-155, which was transferred to other PC cells via exosomes. MiR-155 contributed to resistance among PC cells via anti-apoptosis pathways and suppression of deoxycytidine kinase (dCK), a key gemcitabine-metabolizing enzyme [9, 88]. Furthermore, miR-155 overexpression upregulated the synthesis and secretion of exosomes and miR-155 contents in exosomes [9], which formed a positive loop in regulating GEM resistance. The positive role of EVs in transporting RNA among cancer cells to induce resistance has also been observed in breast and lung cancers [82, 89].

In addition, EVs conferred chemoresistance to PC cells by promoting ROS detoxification through increases in the expression of the ROS detoxifying genes superoxide dismutase 2 (SOD2) and catalase (CAT) [88]. Moreover, CAFs, which were intrinsically resistant, play an active role in GEM resistance by increasing EV release upon exposure to GEM, leading to the upregulation of chemoresistance-inducing factor Snail in recipient PC cells [54]. The expression of Snail and its target, miR-146a, were also upregulated in GEM-treated CAF-derived EVs [54], which may partially explained the increase in Snail expression in recipient cells. GEM increases Snail expression in PC cells [90]; therefore, CAF-derived EVs may increase Snail expression in recipient cells by transferring GEM or its metabolite to PC cells at a dose that is not sufficient to induce cytotoxicity but induces resistance. In the tumor microenvironment, miR-21 derived from macrophages and CAFs are also transferred to cancer cells via EVs, inducing chemoresistance by activating the phosphoinositol 3-kinase (PI3K)/AKT signaling pathway or binding apoptotic peptidase activating factor 1 (APAF1) [83, 85]. Taken together, EVs from PC cells or other cell types in the tumor microenvironment facilitate chemoresistance by regulating RNAs, proteins, relevant genes and signaling pathways, and extensive investigations are required to further explain EV-related chemoresistance in PC.

## EVs as diagnostic and prognostic biomarkers of PC

Due to the non-specific symptoms in early stages, most patients (approximately 80–85%) are diagnosed with metastatic or locally advanced PC at the initial examination [91, 92]. Patients who are diagnosed incidentally during medical examinations have a better prognosis than patients with evident symptoms, and patients with PC diameters smaller than 10 mm can expect a 5-year survival rate of 80.4% [93, 94]. Therefore, methods for the early detection of PC are urgently needed to improve the overall survival of patients with PC. Currently, the most widely used and the only FDA-approved biomarker for PC is CA19–9. However, CA19–9 is not optimal for PC screening because of its relatively low sensitivity and specificity (70–90% and 68–91%, respectively) [95]; it is therefore primarily used to monitor progression and the therapeutic response [94]. More importantly, 5%–10% of the population are Lewis antigen negative and unable to produce CA19–9 [94]. Hence, it is urgent to find new biomarkers. The ideal biomarkers for PC should detect the initial lesions at early stage with high sensitivity and specificity, differentiating PC with healthy ones and benign pancreatic diseases. Besides, they should be able to predict progression and prognosis, contributing to more effective therapy managements. Up to now, extensive investigations have been conducted to identify EVs as novel biomarkers for PC. Because EVs contain specific molecules of original cells, and display stability and abundance in various biological fluids [96], which may lead to a higher sensitivity and specificity in PC diagnosis. Compositions of EVs cover most cancer cell-associated biomarkers, including proteins, mRNAs, miRNAs, and DNA [97]. Proteins and miRNAs are the focus of EV biomarker research.

EVs used for biomarker detection are usually isolated from blood. Researchers applied RT-PCR to analyze the exosomal miRNAs extracted from the blood of 22 individuals with PC (9 at early stage and 13 at advanced stage) and 27 individuals without PC (6 with ampullary carcinoma, 7 with benign pancreatic tumors, 6 with chronic pancreatitis and 8 healthy participants) and assess the roles of four miRNAs (miR-17-5p, miR-21, miR-155 and miR-196a) as biomarkers of PC [98]. Levels of miR-17-5p and miR-21 were elevated in patients with PC, with sensitivity and specificity values of 72.7% and 92.6% and 95.5% and 81.5%, respectively. MiR-17-5p expression was elevated in metastasis and advanced PC, indicating that it was a potential biomarker for unresectable PC [98]. Similar results were reported in the investigations of miR-550 [99] and miR-10b [100], which displayed increased levels in exosomes isolated from the plasma of patients with PC and conditioned media from PC cell lines, suggesting that they may serve as early biomarkers in PC diagnosis.

In addition to miRNAs, EV proteins play a significant role in PC diagnosis. MIF was expressed at high level in plasma exosomes isolated from PC mouse models with liver metastasis compared with healthy ones. Besides, the increased MIF level was also present in mice with PanIN lesions, suggesting that it might serve as a biomarker for early diagnosis and predicting liver metastasis [3]. In 2015, *Nature* published an article presenting a near-perfect diagnostic biomarker for PC – glypican-1 (GPC1) – a cell surface proteoglycan that is specifically enriched on cancer cell-derived exosomes [101]. GPC1^+^ circulating exosomes were significantly elevated in patients with PC (including carcinoma-in-situ, stage I–IV) compared to healthy individuals, indicating that it may act as an biomarker for all stages of PC and aid in distinguishing PC from benign pancreatic disease and healthy individuals, while CA 19–9 levels in the serum fail to distinguish patients with PC from those with benign pancreatic disease. The sensitivity and specificity of GPC1+ circulating exosomes in diagnosing PC were both 100% [101]. Furthermore, the GPC1^+^ exosome level reflected the tumor burden and distant metastasis, and a reduction in the number of GPC1^+^ exosomes was related to increased survival [101]. Interestingly, however, another study indicated that high levels of exosomal miR-10b, miR-21, miR-30c, and miR-181a and low levels of miR-let7a differentiated PC from normal control and chronic pancreatitis samples, while GPC1 level was not significantly different between normal, PC and chronic pancreatitis samples [102]. The difference may be explained by different sample volumes, or different antibodies for GPC1. Except for single EV biomarker, investigators utilized a combination of exosomal proteins and serum/exosomal miRNAs to diagnose PC [103]. In this study, the exosomal protein markers included CD44v6, TSPAN8, epithelial cell adhesion molecule (EpCAM), MET, and CD104, whereas miR-1246, miR-4644, miR-3976, and miR-4306 were selected as exosomal miRNA markers. Concomitant evaluation of these markers exhibited a sensitivity of 1.00 (confidence interval (CI) 0.95–1) and specificity of 0.80 (CI 0.67–0.90) for PC compared with all other groups and a sensitivity of 0.93 (CI 0.81–0.98) when non-PC-malignancies were excluded [103].

In addition to plasma/serum biomarkers, salivary biomarkers have shown potential in diagnosing PC, possibly because miRNA expression profiles in saliva are similar to those in serum [104]. In the study including 12 pancreatobiliary tract cancer patients and 13 healthy donors, salivary exosomal miR-1246 and miR-4644 exhibit great potential as pancreatobiliary tract cancer early biomarkers [105]. Besides, Salivary exosomal proteins are also utilized for the detection of PC.[107]Thus, tests of salivary EV-derived miRNAs and proteins may be a novel method for diagnosing PC.

The extraction of EV biomarkers from body fluids typically requires multiple ultracentrifugation steps, which are time consuming. Several investigators have proposed new technologies to simplify this process. A microfluidic-based platform to isolate circulating exosomes comprised an ExoChip with an antibody against CD63 to capture exosomes and a standard plate-reader for subsequent exosome quantification [107]. An ultrasensitive localized surface plasmon resonance (LSPR)-based microRNA sensor with single nucleotide specificity was developed to quantify microRNA-10b levels, which are elevated in exosomes from the plasma of patients with PC [100]. In addition, another novel microfluidics-based approach was presented by researchers from the University of Notre Dame. This platform consisted of surface acoustic wave (SAW) exosome lysis and ion-exchange nanomembrane RNA sensing performed concurrently on two separate chips; this platform has the advantages of a shorter analysis time (1.5 h for the total analysis), smaller sample volumes (100 μL), and reduced sample loss [99]. Table 1 summarizes the current diagnostic biomarkers for PC.

**Table 1:** EVs biomarkers for pancreatic cancer diagnosis

Biomarker	Sample	Sensitivity and specificity	Methods	Reference
miR-17-5p, miR-21	Serum:22 PCs, 6 benign pancreatic tumors, 7 ampullary carcinomas, 6 CPs, 8 healthy donors	72.7% and 92.6% for miR-17-5p 95.5% and 81.5% for miR-21	Ultracentrifugation for exosome isolation; RT-PCR for miRNA screening	[98]
miR-550	Media from the PANC1 cell line	Not mentioned	SAW for exosomes lysis; ion-exchange nanomembrane sensor for miRNA detection	[99]
miR-10b	Plasma:3 PCs, 3 CPs, 3 healthy donors	Not mentioned	Ultracentrifugation for exosome isolation; LSPR-Based sensor for miRNA quantification	[100]
miR-10b, miR-21, miR-30c, miR-181a, miR-let7a	Blood:29 PCs, 11 CPs, 6 normal donors	Sensitivity:100% Specificity:100% For all these biomarkers	Ultracentrifugation for exosome isolation; RT-qPCR for miRNA detection	[102]
miR-1246, miR-4644, miR-3976, miR-4306 and CD44v6,Tspan8,EpCAM, MET, CD104	Serum: 131 PCs, 25 CPs, 22 benign pancreatic tumors, 12non-PCs, 30 healthy donors	Sensitivity:100% Specificity:80% With 93% for excluding non-Pa-malignancies	Ultracentrifugation for exosome isolation; RT-PCR for miRNA detection; flow-cytometry for protein analysis	[103]
miR-1246, miR-4644	saliva:12 pancreatobiliary tract cancer patients, 13 healthy donors	66.7% and 100% for miR-1246 75.0% and 76.9% for miR-4644	Total Exosome Isolation Reagent for exosoems isolation;RT-qPCR for miRNA detection	[105]
Apbblip, Aspn, BCO31781, Daf2, Foxp1, Gng2,Incenp	Salivary glands from PC mouse model	Not mentioned	Ultracentrifugation for exosome isolation; Western-blotting for protein anaysis	[106]
MIF	Plasma: 5 mice with PanIN, 8 mice with PC, 6 heallthy mice	Not mentioned	ultracentrifugation for exosome isolation; ELISA for MIF measurement	[3]
Glypican-1	Serum: 32 breast cancer, 190 PCs, 100 healthy donor	Sensitivity:100% Specificity:100%	Ultracentrifugation for exosome isolation;ultraperformance liquid chromatography-mass spectrometry(UPLC-MS) for protein evaluation	[101]

Majority of these studies included both patients with benign pancreatic diseases and healthy individuals as controls to assess the role of EV biomarkers for PC diagnosis, and various EV biomarkers proved to indicate disease progression and predict prognosis. However, further studies on EVs biomarkers need to be conducted to assess response to therapy, such as chemotherapy and radiotherapy. Besides, several obstacles remain to be overcome before the clinical application of EVs as biomarkers: prolonged EV isolation procedures, unavailability of specific markers to separate tumor cell-derived EVs from normal cell-derived EVs and lack of sensitive system for large cohorts of clinical samples.

## Potential applications of EVs in treating PC

An increasing number of studies has aimed to apply EVs to PC therapy. The roles of EVs in PC treatment in these studies are divided into three categories: (1) tumor-associated immunity stimulators, (2) drug carriers and (3) therapeutic targets.

### EVs as tumor-associated immunity stimulators

EVs released by tumor cells are known to facilitate immune suppression and tolerance toward cancer cells, thus promoting cancer progression. However, EVs might act as a promising immunity stimulator against tumors. EVs secreted by human DCs induced the activation of CD4^+^ T cells in vitro [108]. Researchers enhanced the immune activity of exosomes isolated from cultured PANC-1 cell supernatants by depleting exosomal miRNAs via lysis and ultrafiltration. Notably, miRNA-depleted exosome proteins increased the tumor-killing capacity of DC/CIK toward PC cells, suggesting that modified PC cell-derived exosomes may be a potential immunotherapeutic approach for PC [72]. In addition to PC, tumor cell-derived EVs also induce anti-tumor immune activity in lymphoma [109], hepatocellular carcinoma [110], and colorectal cancer [111]. Furthermore, some clinical trials have confirmed that cancer progression may be halted by EV-associated immunotherapy [111, 112].

### EVs as drug carriers

EVs carry multiple biological substances and are captured by recipient cells, indicating that they may be an ideal drug delivery tool. Unlike synthetic nanoparticle drug delivery systems, EVs harbor transmembrane or membrane-anchored proteins to enhance endocytosis, which promotes the delivery of their contents [113, 114]. After priming with paclitaxel, mesenchymal stromal cells (MSCs) substantially inhibited the proliferation of the CFPAC-1 pancreatic cell line by secreting EVs containing paclitaxel into the conditioned medium [115]. EVs also deliver curcumin, a turmeric root derivative, to recipient PC cells to promote cytotoxicity in vitro [116]. In addition to common chemotherapeutics, EVs could transport RNA and proteins into recipient cells. According to a newly published article, exosomes that had been engineered to convey a siRNA or short hairpin RNA targeting oncogenic KRAS^G12D^, a common mutation in PC, suppressed cancer in mouse models and significantly increased overall survival [117]. Similarly, the delivery of exosomes carrying the survivin T34A mutant, a survivin blocker, to the MiaPaCa-2 cell line increases gemcitabine sensitivity in PC cells [118]. A novel compound comprising exosomes and staphylococcal enterotoxin B (EXO/SEB) induces the apoptosis of pancreatic cell lines and increases the expression of BAX, BAK and FAS [119]. The advantage of exosomes over liposomes in drug delivery may depend on CD47, a widely expressed integrin-associated transmembrane protein that protects exosomes from clearance by monocytes by binding to CD47 and signal regulatory protein alpha (SIRPα) [117].

### EVs as a therapeutic target

**Fig. 2:**
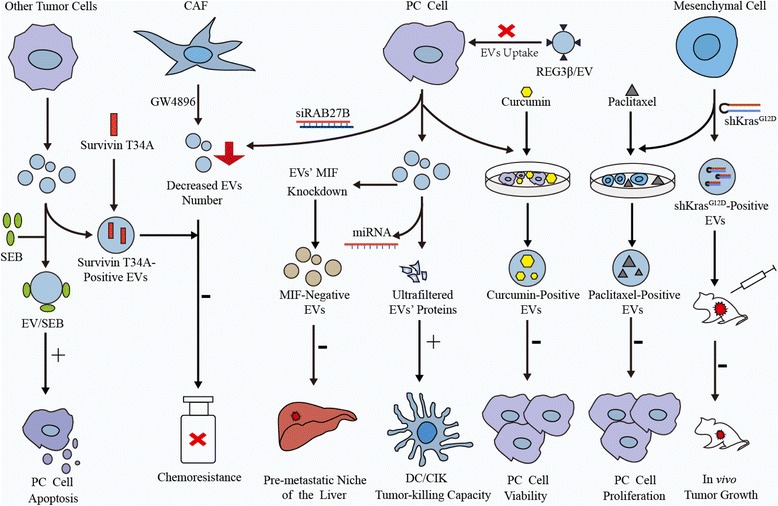
The functions of EVs in PC therapeutic intervention. The potential applications of EVs with different origins in PC therapeutic interventions are shown

As discussed above, EV secretion plays a pivotal role in PC progression and chemoresistance. Thus, strategies that block EV secretion from specific cell types like CAFs or tumor cells may be a potential treatment for PC. The formation of the pre-metastatic niche in the liver was abolished by the silencing of exosomal MIF [3]. Moreover, the chemoresistance of PC cells transferred by GEM-exposed CAFs was eliminated by GW4869, an inhibitor of exosome release, significantly reducing the survival of PC cells [54]. MiR-155-induced GEM resistance was ameliorated by decreasing the number of exosomes following siRAB27B transfection in PC cells [9]. In addition to suppressing EV secretion, inhibition of EV uptake may be another strategy in PC treatment. REG3β, a lectin that binds to EV surface, released by the normal pancreatic tissues surrounding tumor impaired the uptake of EVs by tumor cells both in vitro and *vivo* and inhibited the migration and metabolic changes in cancer cells [120]. All of these studies indicate that EVs may be a potential therapeutic target for the treatment of PC. However, as discussed above, EVs released by different cell types may present pro- or anti- tumor effects. How to block the EV secretion or uptake by specific cell types like CAFs or tumor cells at the exclusion of other cell subsets in tumor microenvironment remains a challenge to overcome. Figure 2 summarizes the functions of EVs in PC therapeutic intervention.

## Conclusions

EVs have various roles in modulating PC progression, chemoresistance, diagnosis and treatment. The underlying mechanisms remain to be further explored, particularly the seemingly contradictory effects of EVs on tumor cell proliferation and tumor-associated immunity. Biomarkers related to exosomes prove to be promising in diagnosing PC and determining patient prognosis; further investigations are required to explore the potential clinical applications of EVs, which may usher in a new era for PC treatment.

## Abbreviations

APAF1: Apoptotic peptidase activating factor 1
CAFs: Cancer-associated fibroblasts
CDEs: CAF-derived exosomes
CIC: Cancer-initiating cell
CIK cells: Cytokine-induced killer cells
CTLs: Cytotoxic T cells
dCK: deoxycytidine kinase
DCs: Dendritic cells
EMT: Epithelial-mesenchymal transition
EpCAM: Adhesion molecule
ESCRT: Endosomal sorting complex required for the transport
EVs: Extracellular vesicles
GEM: Gemcitabine
GPC1: Glypican-1
GSK-3β: Glycogen synthase kinase 3 beta
HES-1: Hairy and enhancer-of-split homolog-1
HLA-DR: Human leukocyte antigen D related
ISEV: The International Society for Extracellular Vesicles
LSPR: Localized surface plasmon resonance
MAPK: Mitogen-activated protein kinase
MDR-1: Multidrug resistant-1
MDSCs: Myeloid-derived suppressor cells
MIF: Migration inhibitory factor
MSCs: Mesenchymal stromal cells
MVBs: Multivesicular bodies
MVs: Microvesicles
NK cells: Natural killer cells
PC: Pancreatic cancer
PDAC: Pancreatic ductal adenocarcinoma
PI3K: Phosphoinositol 3-kinase
PSCs: Pancreatic stellate cells
PTEN: Phosphatase and tensin homolog
RFXAP: Regulatory factor X-associated protein
SAW: Surface acoustic wave
SIRPα: Signal regulatory protein alpha
SNARE: Soluble NSF-attachment protein receptor
SOD2: Superoxide dismutase 2
TAMs: Tumor-associated macrophages
TGF-β: Transforming growth factor β
TLR4: Toll-like receptor 4
